# Socioeconomic status, sport participation, and school-related outcomes among Norwegian adolescents: a cross-sectional analysis

**DOI:** 10.3389/fspor.2025.1613391

**Published:** 2025-08-11

**Authors:** Erik Grasaas, Øyvind Sandbakk, Sergej Ostojic, May Olaug Hovrerak, Tonje Holte Stea

**Affiliations:** ^1^ Teacher Education Unit, University in Agder, Kristi6ansand, Norway; ^2^ School of Sport Science, UiT The Artic University of Norway, Tromsø, Norway.; ^3^ Department of Nutrition and Public Health, Faculty of Health and Sport Sciences, University of Agder, Kristiansand, Norway; ^4^ Division of Health & Social Sciences, NORCE Norwegian Research Centre, Kristiansand, Norway; ^5^ Department of Health and Nursing Science, Faculty of Health and Sport Sciences, University of Agder, Kristiansand, Norway

**Keywords:** adolescence, socioeconomic status, school, sport, physical activity, academic measures

## Abstract

**Background:**

Extensive research highlights the critical role of sport and physical activity (PA) engagement during adolescence, as it is strongly associated with enhanced subjective well-being, reduced risk of mental health problems, and prevention of chronic diseases in adulthood. Thus, it is highly important to identify key barriers of sport participation in adolescence. The present study aimed to; (1) describe socioeconomic status (SES) across different sport disciplines among current participants and sport dropouts, (2) examine possible associations between sport participation and sport dropouts on school-related outcomes.

**Methods:**

School-based cross-sectional data among 90,091 adolescents aged 16–19 years were collected across Norway between 2021 and 2023. A self-report questionnaire was used to assess information about sociodemographic background, current and previous sport participation, and school-related factors, including perceived school stress, teacher care, feeling tired at school and sense of belonging at school. Adjusted binary logistic regressions were conducted using IBM SPSS Statistics.

**Results:**

Adolescents currently participating in sport reported higher SES compared to sport dropouts (2.13 ± 0.51 vs. 1.97 ± 0.61, *p* < 0.01). Tennis participants demonstrated the highest SES of 2.39 ± 0.44, while motorsport participants revealed the lowest score (1.82 ± 0.49). The highest sport attrition rate (80.2%) was revealed among adolescents from the lowest SES level. Current sport participants reported favorable school-related outcomes and PA engagement compared to sport dropouts (all, *p* < 0.01). Adjusted logistic analyses showed that participation in sport activities was associated with lower odds of perceived school stress [OR = 0.83; 95% CI (0.81–0.86)] and being tired during school hours [OR = 0.74; 95% CI (0.71–0.77)], and higher odds for perceived teacher care [OR = 1.17; 95% CI (1.12–1.22)] and perceived sense of belonging [OR = 1.36; 95% CI (1.31–1.42)].

**Conclusions:**

Higher SES was found among adolescents participating in sport compared to sport dropouts, underscoring the importance of promoting affordable sport opportunities during adolescence. Current sport participation was associated with favorable school-related outcomes compared to sport dropouts, such as lower odds for perceived school stress and tiredness in school, and higher odds of perceived sense of belonging in school and perceived teacher care.

## 1 Introduction

Participation in sport and physical activity (PA) during adolescence contributes to numerous positive outcomes including higher subjective well-being, improved cardiovascular health, mental health and academic performance (1–5), yielding longer lifespan and healthier adulthood (6, 7). Despite extensive research evidence which fortifies adolescents' health trough PA and sport participation, most adolescents do not meet the daily recommendation of at least 60 min of moderate-to-vigorous intensity PA per day conveyed by WHO (8). According to Guthold and colleagues, who investigated global PA trends among 1.6 million participants. Findings revealed about 2 out of 10 adolescents adhered to the PA recommendations, with girls being less active than boys (9). A recent Norwegian study revealed similar findings of low adherence to PA recommendations among Norwegian adolescents, also with girls being considerable less active than boys (10). While there are numerous reasons for low PA adherence in adolescence, it is pivotal from a public health perspective to identify and understand barriers to advance the field (11).

According to a systematic review by Stalsberg and Pedersen conducted in 2010, socioeconomic status (SES) could be a relevant barrier, as adolescents from higher-SES families were often more physically active than those from lower-SES families (12). However, the findings were far from uniform, as forty-two percent of the studies included revealed no association or a negative association between SES and PA. Research has also pointed to changes in inequalities over time related to PA and sports participation. A repeated European cross-sectional measures from 2005 to 2019 revealed an overall increase in inequalities (13). Furthermore, other related factors such as demographic factors may also influence adolescents' options for organized sports, due to fewer facilities and options in communities characterized by high proportion of lower SES residents (14). Studies have shown that higher SES levels in adolescence have been linked to higher participation in sport, but also inversely linked to time spent in sedentary behaviors (15). According to Pooja and colleagues, younger US adolescents from high-SES families had three times higher odds of adhering to PA recommendations and three times higher odds of ever participating in sport compared to adolescents from low-SES families (16). Still, the infrastructure of communities and set-up of clubs differ across countries, which should be taken into consideration when addressing sport participation in adolescence (17).

While Norway has a solid welfare system, previous research indicates that participation in organized sport activities is associated with SES (18). Sport participation in Norway is organized through sports clubs, voluntary work, wherein a certain extent of parental involvement is required (19). Therefore, competence, time and resources of parents are usually needed to fulfill the given requirements within the sports disciplines. According to the Norwegian Sports Federation survey, describing cost and cost drivers for youth in 2024, findings revealed a mean annual cost for a 15 year-old cross country skier of 11.700 NOK (equivalent to $1,000) (20). Higher economic demands in sport presumably impact the attrition among lower SES families. These assumptions are interesting to persue, as the Norwegian Social Research (NOVA), have reported that over half of those who engaged in organized sport at beginning of adolescence, had quit by the age of 17 (21). In addition, there exist some evidence of a social gradient for lower inactivity and non-participation in sports among Norwegian adolescents (22). However, further research is needed to identify differences in SES across all sports disciplines in Norway and explore differences between currents sport participants and sport dropouts (no longer participating in their most active sport as of the survey date).

In Norway, there is also lack of knowledge regarding how sport participation may affect school participation and school engagement. The obvious upside of sport participation is higher PA levels, which have been proven robust positive relations to academic performance (23–28). However, these are presumably bidirectional relationships, where better academic engagement might lead to both higher sport participation and teacher attention. There are also reported downsides in young athletes (29). Specifically, sport participation concentrated on one sport during adolescence has shown to increase the risk of burnout, sport-related injuries and attrition (30). Moreover, findings indicate that the positive outcomes of sport participation differ among boys and girls in terms of personal, peer and environmental factors (31), which could impact the perception of school environment as well. PA is shown to enhance adolescents’ psychological resilience and boost self-efficacy, which could be underlying mechanisms explaining a higher sense of belonging in school (32). Hence, accounting for not only SES and PA, but also gender should be considered appropriate when understanding the impact of sport participation and school-related outcomes. The PA engagement within the respective sport disciplines is described elsewhere (33), which could give a broader understanding of sport participation and school outcomes. To clarify, there are research gaps related to identifying key barriers to sport participation and to providing insights into the everyday lives of current sport participants and sport dropouts. Therefore, this study aimed to (1) describe SES across different sport disciplines among current participants and sport dropouts, (2) examine possible associations between sport participation and sport dropouts on school-related factors, such as perceived school stress, perceived teacher care, sense of belonging in school and perceived tiredness in school.

We hypothesized that current sport participants would have higher socioeconomic status (SES) levels than sport dropouts across all sport disciplines and that current sport participation, compared to sport dropout, would be associated with more favorable school-related outcomes.

## 2 Material and methods

### 2.1 Study design and participants

This study utilized cross-sectional data aggregated from the Norwegian Ungdata Survey from 2021 to 2023. The Ungdata study has been conducted annually since 2010. Every year, different Norwegian counties are invited to participate and within a three-year period, all Norwegian counties are represented. According to the Ungdata study, their data provides nationwide information on Norwegian adolescents' health and lifestyle (34). Due to general data protection regulation (GDPR) restrictions, this current dataset, which includes specific sports disciplines, does not include information regarding specific counties, municipalities, schools, ethnicity or age.

The study includes Norwegian adolescents attending 1st to 3rd year of high school, equivalent to an age of 16–19 years. A total of 90,091 adolescents were included in this study, which consisted of current sport participants and sport dropouts. This current dataset included the nineteen following sports: 1. Football, 2. Handball, 3. Basketball, 4. Volleyball, 5. Bandy, 6. Ice-Hockey, 7. Cross-country (XC) skiing, 8. Alpine skiing, 9. Athletics, 10. Swimming, 11. Gymnastics, 12. Dancing, 13. Cheerleading, 14. Tennis, 15. Martial art, 16. Horse-riding, 17. Climbing, 18. Motorsport. 19. Other.

### 2.2 Data collection

The Norwegian Social Research (NOVA) at Oslo Metropolitan University, the regional center for drug rehabilitation (KoRus) and the municipal sector's organization (KS) oversee the Ungdata study, which is financed through the national budget by funds from the Norwegian Directorate of Health (34). The Ungdata survey is an electronic survey conducted in the classroom during a regular school hour. Participation is voluntary and if the adolescents do not choose to participate, they are assigned with regular schoolwork. The Ungdata comprises a mandatory module (for all counties) and an optional module, wherein the counties and municipalities may choose to include additional health-related questions and statements.

### 2.3 Variables

The Ungdata study includes sociodemographic measures and various health-related questions and statements. The following study variables are included in this cross-sectional analysis.

#### 2.3.1 Socioeconomic status

The Ungdata study provides a validated construct for SES (34). The instrument is presented by using a continuous scale from 0.00 to 3.00, whereas 0.00 represents the lowest level of SES and 3.00 the highest level of SES. To provide a natural categorization of low, medium and high SES levels related to attrition rate, the following categories were used: 0.00 to 1.00, 1.00 to 2.00, and 2.00 to 3.00. The validated SES measure in Ungdata stems from the Family Affluence Scale II, which originates from WHO by Currie and colleagues (35, 36). The SES measure includes numerus factors such as the adolescents’ perception of the family economy, parental educational level and level of prosperity.

#### 2.3.2 Current sport participants or sport dropouts

Sport participation was measured by using the following question: “*What sport are you participating in? If you are participating in several sports, choose the one you are most active in”.* The participants could choose from the abovementioned 22 different sport disciplines or the “other” category. Sport dropout (previous sport participation) was measured by the following question: “*What sport did you quit? If you are involved in several sports, choose the one you most recently participated in.* The participants could choose from the same sport disciplines or the “other” category. This question is included in numerous Ungdata collection waves, but not formally validated. In addition, due to low number of participants attending certain sports activities, such as lacrosse, snowboarding, cricket and golf, participation in these specific sports disciplines were merged by the distributor into the “other” category.

#### 2.3.3 Physical activity levels

PA levels were assessed using the question, “*How often are you so physically active that you become short of breath or sweaty?*”. Adolescents could choose from six response alternatives: “*never active”, “rarely”, “1*–*2 times a month”, “1*–*2 times a week”, “3*–*4 times a week”,* and “*at least 5 times a week”.* These respective categories were merged and dichotomized, whereas the latter two categories represented “*PA several times a week”.* Due to the mandatory weekly physical education among these school-based adolescents, “PA several times a week” was considered the most appropriate dichotomization to assess relevant differences between groups. Independent PA training was measured using the following question: “*Do you exercise or train on your own” (run, swim, cycle or walk).* The participants could choose from the same six response alternatives, whereas the latter three and first three categories were merged into “*weekly independent PA”* or not, respectively. Single-item measures of PA have previously shown considerable validity and reliability (37), making them indispensable in contexts settings when device-based measurements are impracticable (38).

#### 2.3.4 School-related outcomes

Perceived school stress was measured by using the statement “*I get stressed by schoolwork”.* Participants could choose from five response alternatives that were provided, “*never”, “seldom”, “sometimes”, “often”* and “*very often”*. The variable was dichotomized, wherein the latter two categories were recoded as “*often perceived school stress”.* Psychological variables from the Nordic countries have shown that a single-item stress symptom measure demonstrated satisfactory content, criterion and construct validity (39). Perceived teacher care was assessed by the statement “My teachers care about me.” The statement had four response alternatives: “*totally agree”, “somewhat agree”, “somewhat disagree”,* and *“totally disagree”,* wherein agreeing to the statements was recoded as “*High perceived teacher care”.* Sense of belonging in school was measured by the statement: “*I feel like I fit in with the pupils at school”.* The statement had four response alternatives*: “totally agree”, “somewhat agree”,* “*somewhat disagree”,* and “*totally disagree”,* wherein agreeing to the statements was recoded as “*High sense of belonging in school”.* Perceived tiredness in school was assessed with the statement: “*I have been so sleepy/tired that it has affected school or leisure activities”.* The participants could choose from the following response alternatives: “*No days” “1*–*2 days” “3*–*4 days”* or “*5 days or more”.* The two latter categories represented “*Often perceived tiredness in school”.* The school-related variables are used in several data collection waves in Ungdata and “Young in Oslo”, but not formally validated. The school-related outcomes are treated as dichotomous variables in the analysis.

### 2.4 Ethical consideration

All participation in the Ungdata survey is voluntary. As the adolescents were 16 years and older, they were allowed to make independent decision regarding consenting to participation (parental consent not needed). Informed written consent was obtained from all participating adolescents. The included question in the Ungdata survey has been approved by the Norwegian Agency for Shared Services in Education and Research (ref. 821474), known as SIKT (40). To receive data regarding specific organized sport, an additional application was sent to The Norwegian Social Research (NOVA) at Oslo Metropolitan University. The application was approved (ref. 24–22), however due to GDPR and privacy regulations, this current study does not possess all the variables as otherwise are accessible in the national dataset. This current study is reported in accordance to the Strengthening the Reporting of Observational Studies in Epidemiology (STROBE) guidelines (Supplementary File 1) (41).

### 2.5 Statistical analyses

All statistical analyses were performed using IBM SPSS Statistics for Mac, Version 29.0 (IBM Corp., Armonk, NY, USA). Descriptive measures for continuous variables are presented as means and standard deviations (SDs). Chi-square tests and independent *t*-test were conducted to test differences in study variables between adolescents currently participating in sport and sport dropouts. Binary logistic regression analyses were conducted to estimate the associations between the independent dichotomized variable (current or former participant of sport) and the dichotomized dependent school-related outcome variables by controlling for socioeconomic status and PA levels, stratified by gender. Regressions are presented with beta coefficient with 95% confidence intervals with *p*-values < 0.05 considered statistically significant. Given the large sample size and high response rate, neither bootstrapping or imputation was employed.

## 3 Results

### 3.1 Participants

A total of 90,091 Norwegian adolescents participated in this study, of which 67.9% (*N* = 61,151) were sport dropouts and 32.1% (*N* = 28,940) current sport participants. Among the sport dropouts, 55.8% were girls (*N* = 33,509) and 44.2% were boys (*N* = 26,587), whereas for the adolescents currently participating in sport, 54.2% were boys (*N* = 15,490) and 45.8% were girls (*N* = 13,110). The included study variables revealed a high response rate (>98%, Appendix 1), except the variable related to sleep, which was part of the optional module in Ungdata (67.7%).

### 3.2 Descriptive statistics

Adolescents currently participating in sport reported higher SES across all disciplines compared to those who had dropped out of the respective sport, except for participants in motorsport (mean/SD 1.83 ± 0.50) vs. 1.82 ± 0.49). Tennis participants demonstrated the highest SES, with a mean/SD score of 2.39 ± 0.44 on a 0.00–3.00 scale, while motorsport participants showed the lowest mean score (Table 1).

**Table 1:** Socioeconomic status across sports disciplines expressed as mean/SD.

Sport disciplines	Previous participants	Counts (% of total)	Current participants	Counts (% of total)
Football	1.93 (0.55)	26,648 (43.6%)	2.09 (0.52)	12,232 (42.3%)
Handball	1.98 (0.54)	10,001 (16.4%)	2.16 (0.47)	4,783 (16.6%)
Basketball	1.97 (0.61)	1,220 (2.0%)	2.08 (0.57)	637 (2.2%)
Volleyball	1.95 (0.57)	1,321 (2.2%)	2.15 (0.51)	1,161 (4.0%)
Bandy	2.00 (0.55)	588 (1%)	2.18 (0.50)	246 (0.9%)
Ice hockey	1.99 (0.53)	413 (0.7%)	2.07 (0.50	384 (1.3%)
XC skiing	2.23 (0.50)	796 (1.3%)	2.33 (0.42)	804 (2.8%)
Alpine skiing	2.25 (0.48)	274 (0.4%)	2.27 (0.44)	189 (0.7%)
Athletics	2.11 (0.53)	1,418 (2.3%)	2.28 (0.48)	631 (2.2%)
Swimming	2.05 (0.53)	2,028 (3.3%)	2.21 (0.48)	556 (1.9%)
Gymnastics	1.98 (0.54)	3,323 (5.4%)	2.15 (0.50)	651 (2.3%)
Dancing	2.04 (0.55)	3,304 (5.4%)	2.23 (0.47)	734 (2.5%)
Cheerleading	1.99 (0.55)	470 (0.8%)	2.10 (0.53)	178 (0.6%)
Tennis	2.28 (0.48)	669 (1.1%)	2.39 (0.44)	324 (1.1%)
Martial art	1.93 (0.56)	3,012 (4.9%)	2.06 (0.53)	1,156 (4.0%)
Climbing	2.17 (0.48)	484 (0.8%)	2.29 (0.46)	240 (0.8%)
Horse-riding	1.97 (0.53)	1,159 (1.9%)	2.08 (0.49)	925 (3.2%)
Motorsport	1.83 (0.50)	335 (0.5%)	1.82 (0.49)	296 (1.0%)
Other sports	1.99 (0.54)	3,594 (5.9%)	2.17 (0.51)	2,767 (9.6%)

**Figure 1:**
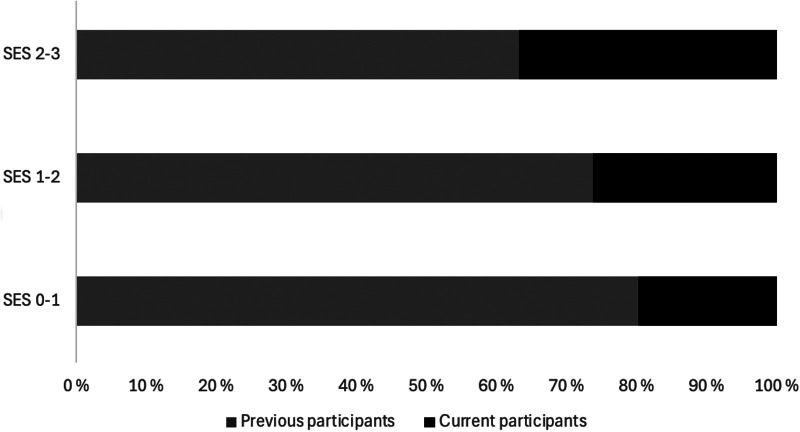
SES scores for the total sample stratified by previous participants and current participants in sports and by SES levels; 2-3 indicates the highest levels (*N* = 51,265), 1-2 middle SES level (*N* = 36,410), and 0-1 the lowest SES level (*N* = 2,264).

The trend of lower SES levels among sport dropouts is depicted in Figure 1, illustrated by a stacked area plot of the respective SES categories (0–3). Among adolescents from lowest SES levels (0–1), about four out of five adolescents dropped out of sport (80.2%). Whereas adolescents from middle SES levels, about three out of four dropped out (73.7%). While about two out of three (63.2%) of the adolescents from the highest levels of SES dropped out, underscoring the relatively higher retention levels among adolescents from higher SES levels and the worrying drop-out trend related to adolescents from lower SES categories.

### 3.3 Comparison between current sport participants and sport dropouts

Higher SES mean score was revealed among adolescents participating in sport compared to sport dropouts (*p* < 0.01). Favorable school-related outcomes were unveiled among adolescents currently participating in sport (Table 2), by higher perception of teacher care, sense of belonging of the school environment and lower perceived school stress and less tiredness in school compared to sport dropouts (all *p* < 0.01). Higher PA levels were revealed among adolescents participating in sport compared to sport dropouts in addition to higher weekly independent training such as running, cycling, swimming or walking (both *p* < 0.01).

**Table 2:** Study variables stratified by previous participants and current participants in sports expressed as mean/SD.

Study variables	Previously participated	Currently participating
SES (mean/SD)	1.97 (0.61)	2.13 (0.51)*
Often perceived school stress	56.0%	50.0%*
High perceived teacher care	82.9%	85.5%*
High perceived sense of belonging	80.9%	87.3%*
Often perceived tiredness in school	32.4%	23.8%*
Weekly independent PA	39.3%	45.9%*
PA several times a week	42.9%	82.4%*

* *p* < 0.01, *t*-test for continuous variable (SES) and chi-square tests used to compare groups.

### 3.4 Associations between sport participation and school-related outcomes

Regressions revealed that being currently active in sport rather than being previously active were associated with favorable school-related outcomes (Table 3), including lower odds ratio (OR) for perceived school stress [OR = 0.79; 95% CI (0.77–0.81)], and tiredness in school [OR = 0.65; 95% CI (0.63–0.68)], higher odds for perceived teacher care [OR = 1.21; 95% CI (1.16–1.26)] and higher perceived sense of belonging of the school environment [OR = 1.63; 95% CI (1.56–1.69)]. Associations remained significant after adjusting for socioeconomic status and PA levels (all *p* < 0.01).

**Table 3:** Binary logistic regressions between sport participation or not (independent variable) and school-related outcomes (dependent variables) for the total sample.

Total sample	School stress	Teacher’ support	Sense of belonging at school	Tiredness in school
	OR (95% CI)	OR (95% CI)	OR (95% CI)	OR (95% CI)
Crude				
Current sport participants (ref)	1	1	1	1
Sport dropouts	0.79 (0.77–0.81)**	1.21 (1.16–1.26)**	1.63 (1.56–1.69)**	0.65 (0.63–0.68)**
Adjusted*				
Current sport participants (ref)	1	1	1	1
Sport dropouts	0.83 (0.81–0.86)**	1.17 (1.12–1.22)**	1.36 (1.31–1.42)**	0.74 (0.71–0.77)**

Adjusted for socioeconomic status and physical activity levels, *p < 0.05, **p < 0.01.

Adjusted regressions stratified by gender revealed that sport participation was associated with favorable school outcomes for boys and girls (Table 4). Being a current sport participant revealed twice as high odds for high perceived sense of belonging of the school environment among boys (38%) than in girls (19%), after adjusting for SES and PA. While girls in sports reported twice the odds of higher perceived teacher care [OR = 1.18; 95% CI (1.11–1.25)] than boys [OR = 1.09; 95% CI (1.02 to 1.15)] and over twice the lower odds for perceived school stress [OR = 0.84; 95% CI (0.80–0.88)] than boys [OR = 0.94; 95% CI (0.89–0.98)].

**Table 4:** Adjusted binary logistic regressions between sport participation or not (independent variable) and school-related outcomes (dependent variables) for boys and girls.

Gender	School stress	Teacher’ support	Sense of belonging at school	Tiredness in school
	OR (95% CI)	OR (95% CI)	OR (95% CI)	OR (95% CI)
Girls				
Current sport participants (ref)	1	1	1	1
Sport dropouts	0.84 (0.80–0.88)**	1.18 (1.11–1.25)**	1.19 (1.12–1.26)**	0.76 (0.72–0.81)**
Boys				
Current sport participants (ref)	1	1	1	1
Sport dropouts	0.94 (0.89–0.98)**	1.09 (1.02–1.15)**	1.38 (1.30–1.48)**	0.80 (0.75–0.85)**

Adjusted for socioeconomic status and physical activity levels, *p < 0.05, **p < 0.01.

## 4 Discussion

The aims of the current study were to describe SES across sport disciplines in current sport participants and in sport dropouts and to examine possible associations between sport participation and sport dropouts on school-related outcomes. The main findings were as follows: (1) We found higher SES among adolescents participating in sport compared to sport dropouts and (2) participation in sport was associated with favorable school-related outcomes, such as lower odds for perceived school stress and tiredness in school, and higher odds of perceived sense of belonging in school and perceived teacher care.

Results from the present study show that current sport participants had higher socioeconomic status (SES) levels than sport dropouts across all sport disciplines, except for adolescents in motorsports. Our findings indicating that participants in tennis and XC skiing have higher SES levels that participants in other sports aligns with results from a repeated cross-sectional study among adults (13). These findings indicate that historical and cultural aspects related to sport participation and socioeconomic class may to some extent preserved to this day in Norway. Most worrying is our findings showing highest attrition rate among adolescents with the lowest SES levels. Barriers to PA among adolescents are often reported due to the lack of time, lack of motivation, and lack of accessible places (42). On the other hand, a systematic review of qualitative studies reported the main PA barriers and facilitators in the following order: individual factors, social and relational factors, PA nature, life factors and sociocultural and environmental factors (43). However, in the systematic review and meta-analyses by Owen colleagues (44), investigating the socioeconomic disparity in PA between low and high SES households, found even greater disparity in children than in adolescents. To elucidate, there is a need for concerted efforts to accommodate the complex need and barriers in an early life phase.

While our results revealed favorable school-outcomes and PA among adolescents participating in sport compared to sport dropouts, the picture is complex with several underlying conditions. Besides differences in SES, clear contrasts in PA engagement between groups should be highlighted, as sport participation and higher PA engagement presumably have complementary roles. While PA provide a broad range of physiological beneficial responses that could be beneficial in a school setting such as enhancing cognitive function, memory and attention (45–47), sport participation presumably provides structural long-term engagement, collaboration with peers and goal setting, which are also transferable to a school context (2, 48). Hence, both PA engagement and sport participation seem to mutually reinforce their positive impact on academic outcomes. Moreover, it is interesting that among adolescents currently participating in sport, fewer reported tiredness in school compared to sport dropouts, despite several mandatory practices during the weekdays, often late at night. These findings align with previous research revealing overall better health-related outcomes among adolescent in sport vs. adolescents not in organized sport, despite sport participants even reported less sleep duration than adolescents not active in sport (49).

Despite robust associations of favorable school outcomes among current sport participants for the total sample, which aligns with current research evidence underscoring the importance of PA engagement for positive relations to academic performance (23–28). It is interesting to discuss the nuances according to gender. Being a current sport participant revealed twice as high odds for higher perceived sense of belonging of the school environment among boys than in girls. These findings are presumably attributed by social and cultural aspects in adolescence. Boys in adolescence tend to be more competitive in sport compared to girls and physical competitiveness and capacities among boys are socially linked to a source of pride and admiration (50, 51). Therefore, the cultural valuation of being a sport participant can in some cases be boys-dominated and provide more explicit status. However, being a sport participant among girls and seems to be more strongly associated with lower odds of perceived school stress, which might indicate that sport participation, especially in girls is an essential arena for building resilience and capacities that could reduce the feeling of stress, which is a key finding as girls tend to report more perceived school stress compared to boys during adolescence (52, 53). In addition, girls reported higher odds of perceived teacher care than boys, which may presumably be explained to some extend by larger vocabulary (54) and often more openness regarding emotional struggles and concerns among girls than boys (55). These findings are in accordance with the research literature, revealing that girls tend to be more active in the classrooms and higher engagement in teacher-student relations (56–58) regardless of sport participation.

### 4.1 Implications

This paper expands upon previous research by identifying SES levels across sport disciplines and uncovering the highest attrition rate among adolescents from the lowest SES levels and further provide insight into how sport participation impacts academic outcomes, while adjusting for relevant covariates. Although high dropout rates from sport activities during adolescence are expected, high attrition rates among low SES groups are especially concerning as they face additional challenges that may partly be reduced due to the positive impact participating in PA may have in tackling everyday life in this critical life phase. In the period ahead, policymakers and decision-makers should prioritize coordinated efforts to facilitate sport participation, particularly among adolescents from lower socioeconomic backgrounds. Such targeted strategies are essential for promoting equity in access to PA. Furthermore, future observational and experimental research is needed to better understand the mechanisms underlying participation disparities and to evaluate the effectiveness of intervention strategies.

### 4.2 Strengths and limitations

Several key factors have contributed to the study's credibility. The study benefits from using a sample aggregated from all parts of Norway, which increases the validity and representativeness of the findings. Moreover, the high response rate and large sample size should be considered a strength. Further, the Ungdata study have a stringent and rigor procedure of data cleaning (34), which identifies and excludes unserious responses. These well-established procedures mitigate corrupt data and thereby increase the study's credibility (60). Finally, by following the STROBE guidelines, this study provides structural transparency in the reporting, which enhances the reliability of the study.

Although data were aggregated over time, this study is using a cross-sectional design, which does not provide any causal interactions nor understanding of trends over time. In addition, cross-sectional study design relies on self-reported data, which introduces potential biases such as like recall and social desirability bias. Recall bias and social desirability bias could be relevant even though the adolescents reported their PA levels and school-related factors on a weekly basis, as some might for instance overreport their PA levels to better fit in (59). Moreover, as we needed to exclude certain demographic variables (e.g., age, ethnicity, municipality) due to GDPR restrictions, this limits the ability to control for important confounders. Further, the variable tiredness in school had a lower response rate (67.7%), which should be consider a limitation. Grouping certain sports under the broad “Other” category due to small sample sizes may mask sport-specific differences. One major limitation is that key measures such as PA, and school-related outcomes, rely on single-item self-reports, which lack formal validation. Although the interpretation of data could be easier to convey with dichotomization of categoric data, it reduces the statistical power and obscures more nuanced relationships, which should be considered a limitation. Adolescents currently participating in sport may be more engaged or interested in questions related to PA compared to sport dropouts, which could introduce response bias and should therefore be considered a limitation of the study. Due to the limitation in the dataset, we were not able to assess the duration or intensity of previous participation nor reasons for quitting, or whether individuals transitioned to other forms of activity. Clearer insight in abovementioned aspects would provide a more holistic picture of dropout's pattern among Norwegian adolescents. The rationale for dichotomize was underpinned by the need for clear interpretation of regressions analyses across school-related outcome variables, as it simplifies the implications of findings to a broader readership, such as teacher, trainers, stakeholders and decision makers. Considering the large sample size and statistical strength, we allowed us to focus on the general positive or negative response (e.g., agree vs. disagree) rather than minor variations of agreement of disagreement. However, dichotomization may mask nuances and could therefore be considered a limitation. In this study period, the pandemic restrictions may have disproportionately affected adolescents' sport participation, SES and school-related factors. Despite the universal restrictions in school, adolescents from lower SES backgrounds may have faced greater barrier in sport participation and school, due to less access to resources in their home setting. These contextual factors may limit the generalizability of our findings to non-pandemic periods.

## 5 Conclusions

In conclusion, this study unveiled higher SES among adolescents in sport than in sport dropouts, underscoring the importance of promoting accessible and affordable sport opportunities during adolescence. Current sport participation was associated with favorable school-related outcomes compared to sport dropouts, such as lower odds for perceived school stress and tiredness in school, and higher odds of perceived sense of belonging in school and perceived teacher care. This indicates a positive role of sport participation in fostering positive school experiences which should be considered when policymakers prioritize future strategies. It is concerning that the highest attrition rate was uncovered among adolescents derived from the lowest SES levels. Thus, future observational and experimental studies are suggested, with the hope of focusing on concerted efforts to reduce barriers for sport participation and PA engagement in adolescence.

## Data Availability

Publicly available datasets were analyzed in this study. This data can be found here: Data supporting the results of this study is available upon request from the Norwegian Agency for Shared Services in Education and Research (SIKT) and NOVA (40). Reference to dataset from SIKT: [https://doi.org/10.18712/NSD-NSD3157-V1]. Request to access these datasets should be directed to https://sikt.no and https://www.ungdata.no/den-nasjonale-databasen/.

## Appendix 1

Sample size and response rate in study variables.

**Table:**

Study variable	N	Response rate
SES	89,939	99.8%
School stress	89,819	99.7%
Teacher care	89,199	99.0%
Sense of belonging	89,025	98.8%
Tiredness in school	60,982	67.7%
Leisure time PA	88,321	98.0%
PA levels	89,598	99.5%
Gender	88,696	98.5%

## References

[1] Santana CCA Azevedo LB Cattuzzo MT Hill JO Andrade LP Prado WL. Physical fitness and academic performance in youth: a systematic review. Scand J Med Sci Sports. (2017) 27(6):579–603. 10.1111/sms.12773 27714852

[2] Eime RM Young JA Harvey JT Charity MJ Payne WR. A systematic review of the psychological and social benefits of participation in sport for children and adolescents: informing development of a conceptual model of health through sport. Int J Behav Nutr Phys Act. (2013) 10(1):98. 10.1186/1479-5868-10-98 23945179 PMC3751802

[3] Dale LP Vanderloo L Moore S Faulkner G. Physical activity and depression, anxiety, and self-esteem in children and youth: an umbrella systematic review. Ment Health Phys Act. (2019) 16:66–79. 10.1016/j.mhpa.2018.12.001

[4] Janssen I LeBlanc AG. Systematic review of the health benefits of physical activity and fitness in school-aged children and youth. Int J Behav Nutr Phys Act. (2010) 7(1):40. 10.1186/1479-5868-7-40 20459784 PMC2885312

[5] Singh A Uijtdewilligen L Twisk JW van Mechelen W Chinapaw MJ. Physical activity and performance at school: a systematic review of the literature including a methodological quality assessment. Arch Pediatr Adolesc Med. (2012) 166(1):49–55. 10.1001/archpediatrics.2011.716 22213750

[6] Högström G Nordström A Nordström P. Aerobic fitness in late adolescence and the risk of early death: a prospective cohort study of 1.3 million Swedish men. Int J Epidemiol. (2016) 45(4):1159–68. 10.1093/ije/dyv321 26686843

[7] Resnick MD Catalano RF Sawyer SM Viner R Patton GC. Seizing the opportunities of adolescent health. Lancet. (2012) 379(9826):1564–7. 10.1016/S0140-6736(12)60472-3 22538176

[8] WHO. *Physical Activity*. (2022) (Accessed 09.04.2024).

[9] Guthold R Stevens GA Riley LM Bull FC. Global trends in insufficient physical activity among adolescents: a pooled analysis of 298 population-based surveys with 1·6 million participants. Lancet Child Adolesc Health. (2020) 4(1):23–35. 10.1016/S2352-4642(19)30323-2 31761562 PMC6919336

[10] Grasaas E Sandbakk Ø. Adherence to physical activity recommendations and associations with self-efficacy among Norwegian adolescents: trends from 2017 to 2021. Front Public Health. (2024) 12:1382028. 10.3389/fpubh.2024.1382028 38846610 PMC11155692

[11] Atkin AJ van Sluijs EMF Dollman J Taylor WC Stanley RM. Identifying correlates and determinants of physical activity in youth: how can we advance the field? Prev Med. (2016) 87:167–9. 10.1016/j.ypmed.2016.02.040 26940254 PMC4893019

[12] Stalsberg R Pedersen AV. Effects of socioeconomic status on the physical activity in adolescents: a systematic review of the evidence. Scand J Med Sci Sports. (2010) 20(3):368–83. 10.1111/j.1600-0838.2009.01047.x 20136763

[13] Richard V Piumatti G Pullen N Lorthe E Guessous I Cantoreggi N Socioeconomic inequalities in sport participation: pattern per sport and time trends—a repeated cross-sectional study. BMC Public Health. (2023) 23(1):785. 10.1186/s12889-023-15650-7 37118691 PMC10141913

[14] Powell LM Slater S Chaloupka FJ Harper D. Availability of physical activity–related facilities and neighborhood demographic and socioeconomic characteristics: a national study. Am J Public Health. (2006) 96(9):1676–80. 10.2105/AJPH.2005.065573 16873753 PMC1551946

[15] Molina-García J Queralt A Adams MA Conway TL Sallis JF. Neighborhood built environment and socio-economic status in relation to multiple health outcomes in adolescents. Prev Med. (2017) 105:88–94. 10.1016/j.ypmed.2017.08.026 28863871

[16] Tandon PS Kroshus E Olsen K Garrett K Qu P McCleery J. Socioeconomic inequities in youth participation in physical activity and sports. Int J Environ Res Public Health. (2021) 18(13):6946. 10.3390/ijerph18136946 34209544 PMC8297079

[17] Balish SM Rainham D Blanchard C. Community size and sport participation across 22 countries. Scand J Med Sci Sports. (2015) 25(6):e576–81. 10.1111/sms.12375 25487738

[18] Andersen PL Bakken A. Social class differences in youths’ participation in organized sports: what are the mechanisms? Int Rev Sociol Sport. (2019) 54(8):921–37. 10.1177/1012690218764626

[19] Jacobsen SE Aandersen PL Nordø ÅD Sletten MA Arnesen D. Sosial ulikhet i barn og unges deltakelsei organiserte fritidsaktiviteter, in Betydningen av sosioøkonomiske ressurser, geografi og landbakgrunn, S.f.f.p.s.o.f. sektor, Editor. (2021).

[20] Norges Idrettsforbund. Kostnader og kostnadsdrivere for barne- og ungdomsidretten. Norges Idrettsforbund, Deloitte (2024). Available online at: https://www.idrettsforbundet.no/contentassets/a4744409156046f9bd0ee569a7f8965a/rapport-kostnader-og-kostnadsdrivere-for-barne--og-ungdomsidretten-2024.pdf (Accessed December 28, 2024).

[21] Strandbu ABÅ, Idrettsdeltakelse blant ungdom—før, under og etter koronapandemien: VELFERDSFORSKNINGSINSTITUTTET NOVA. p. s.49. (2023).

[22] Heradstveit O Haugland S Hysing M Stormark KM Sivertsen B Bøe T. Physical inactivity, non-participation in sports and socioeconomic status: a large population-based study among Norwegian adolescents. BMC Public Health. (2020) 20(1):1010. 10.1186/s12889-020-09141-2 32590961 PMC7318733

[23] Booth JN Leary SD Joinson C Ness AR Tomporowski PD Boyle JM Associations between objectively measured physical activity and academic attainment in adolescents from a UK cohort. Br J Sports Med. (2014) 48(3):265–70. 10.1136/bjsports-2013-092334 24149097 PMC3913217

[24] Maher C Lewis L Katzmarzyk PT Dumuid D Cassidy L Olds T. The associations between physical activity, sedentary behaviour and academic performance. J Sci Med Sport. (2016) 19(12):1004–9. 10.1016/j.jsams.2016.02.010 26971300

[25] Haapala EA Haapala HL Syväoja H Tammelin TH Finni T Kiuru N. Longitudinal associations of physical activity and pubertal development with academic achievement in adolescents. J Sport Health Sci. (2020) 9(3):265–73. 10.1016/j.jshs.2019.07.003 32444151 PMC7242213

[26] Sneck S Viholainen H Syväoja H Kankaapää A Hakonen H Poikkeus AM Effects of school-based physical activity on mathematics performance in children: a systematic review. Int J Behav Nutr Phys Act. (2019) 16(1):109. 10.1186/s12966-019-0866-6 31752903 PMC6873534

[27] Norris E van Steen T Direito A Stamatakis E. Physically active lessons in schools and their impact on physical activity, educational, health and cognition outcomes: a systematic review and meta-analysis. Br J Sports Med. (2020) 54(14):826–38. 10.1136/bjsports-2018-100502 31619381

[28] Solberg RB Steene-Johannessen J Anderssen SA Ekelund U Säfvenbom R Haugen T Effects of a school-based physical activity intervention on academic performance in 14-year old adolescents: a cluster randomized controlled trial—the school in motion study. BMC Public Health. (2021) 21(1):871. 10.1186/s12889-021-10901-x 33957895 PMC8101111

[29] Merkel DL. Youth sport: positive and negative impact on young athletes. Open Access J Sports Med. (2013) 4:151–60. 10.2147/OAJSM.S33556 24379720 PMC3871410

[30] Gonçalves CE Rama LM Figueiredo AB. Talent identification and specialization in sport: an overview of some unanswered questions. Int J Sports Physiol Perform. (2012) 7(4):390–3. 10.1123/ijspp.7.4.390 22868280

[31] Hopkins CS Hopkins C Kanny S Watson A. A systematic review of factors associated with sport participation among adolescent females. Int J Environ Res Public Health. (2022) 19(6):3353. 10.3390/ijerph19063353 35329041 PMC8950299

[32] Peng B Chen W Wang H Yu T. How does physical exercise influence self-efficacy in adolescents? A study based on the mediating role of psychological resilience. BMC Psychol. (2025) 13(1):285. 10.1186/s40359-025-02529-y 40119462 PMC11927186

[33] Grasaas E Sandbakk Ø. Life satisfaction across sports disciplines and sports categories among Norwegian adolescents: comparisons to national data. Front Psychol. (2025) 16:1577326. 10.3389/fpsyg.2025.1577326 40547591 PMC12179080

[34] Bakken A Frøyland LR Sletten MA. Sosiale forskjeller i unges liv. Hva sier Ungdata-undersøkelsene? NOVA Rapport 3/2016. (2016).

[35] Currie C Molcho M Boyce W Holstein B Torsheim T Richter M. Researching health inequalities in adolescents: the development of the health behaviour in school-aged children (HBSC) family affluence scale. Soc Sci Med. (2008) 66(6):1429–36. 10.1016/j.socscimed.2007.11.024 18179852

[36] Currie CE Elton RA Todd J Platt S. Indicators of socioeconomic status for adolescents: the WHO health behaviour in school-aged children survey. Health Educ Res. (1997) 12(3):385–97. 10.1093/her/12.3.385 10174221

[37] Milton K Bull FC Bauman A. Reliability and validity testing of a single-item physical activity measure. Br J Sports Med. (2011) 45(3):203–8. 10.1136/bjsm.2009.068395 20484314

[38] O’Halloran P Kingsley M Nicholson M Staley K Randle E Wright A Responsiveness of the single item measure to detect change in physical activity. PLoS One. (2020) 15(6):e0234420. 10.1371/journal.pone.0234420 32584830 PMC7316312

[39] Elo AL Leppänen A Jahkola A. Validity of a single-item measure of stress symptoms. Scand J Work Environ Health. (2003) 29(6):444–51. 10.5271/sjweh.752 14712852

[40] Norwegian Agency for Shared Services in Education and Research (SIKT). Available online at: https://sikt.no/en/home (Accessed May 10, 2023).

[41] von Elm E Altman DG Egger M Pocock SJ Gøtzsche PC Vandenbroucke JP. The strengthening the reporting of observational studies in epidemiology (STROBE) statement: guidelines for reporting observational studies. Ann Intern Med. (2007) 147(8):573–7. 10.7326/0003-4819-147-8-200710160-00010 17938396

[42] Ferreira Silva RM Mendonça CR Azevedo VD Raoof Memon A Noll P Noll M. Barriers to high school and university students’ physical activity: a systematic review. PLoS One. (2022) 17(4):e0265913. 10.1371/journal.pone.0265913 35377905 PMC8979430

[43] Martins J Costa J Sarmento H Marques A Farias C Onofre M Adolescents’ perspectives on the barriers and facilitators of physical activity: an updated systematic review of qualitative studies. Int J Environ Res Public Health. (2021) 18(9):4954. 10.3390/ijerph18094954 34066596 PMC8125166

[44] Owen KB Nau T Reece LJ Bellew W Rose C Bauman A Fair play? Participation equity in organised sport and physical activity among children and adolescents in high income countries: a systematic review and meta-analysis. Int J Behav Nutr Phys Act. (2022) 19(1):27. 10.1186/s12966-022-01263-7 35303869 PMC8932332

[45] Hallal PC Victora CG Azevedo MR Wells JC. Adolescent physical activity and health: a systematic review. Sports Med. (2006) 36(12):1019–30. 10.2165/00007256-200636120-00003 17123326

[46] Kumar B Robinson R Till S. Physical activity and health in adolescence. Clin Med (Lond). (2015) 15(3):267–72. 10.7861/clinmedicine.15-3-267 26031978 PMC4953112

[47] Bailey R Hillman C Arent S Petitpas A. Physical activity: an underestimated investment in human capital? J Phys Act Health. (2013) 10(3):289–308. 10.1123/jpah.10.3.289 23620387

[48] Wilson OWA Whatman C Walters S Keung S Enari D Rogers A The value of sport: wellbeing benefits of sport participation during adolescence. Int J Environ Res Public Health. (2022) 19:8579. 10.3390/ijerph19148579 35886430 PMC9324252

[49] Yman J Helgadóttir B Kjellenberg K Nyberg G. Associations between organised sports participation, general health, stress, screen-time and sleep duration in adolescents. Acta Paediatr. (2023) 112(3):452–9. 10.1111/apa.16556 36209496 PMC10092197

[50] Deaner RO Geary DC Puts DA Ham SA Kruger J Fles E A sex difference in the predisposition for physical competition: males play sports much more than females even in the contemporary U.S. PLoS One. (2012) 7(11):e49168. 10.1371/journal.pone.0049168 23155459 PMC3498324

[51] Buser T Niederle M Oosterbeek H. Gender, competitiveness, and career choices *. Q J Econ. (2014) 129(3):1409–47. 10.1093/qje/qju009

[52] Grasaas E Skarstein S Mikkelsen HT Småstuen MC Rohde G Helseth S The relationship between stress and health-related quality of life and the mediating role of self-efficacy in Norwegian adolescents: a cross-sectional study. Health Qual Life Outcomes. (2022) 20(1):162. 10.1186/s12955-022-02075-w 36482450 PMC9733140

[53] Högberg B Strandh M. Temporal trends and inequalities in school-related stress in three cohorts in compulsory school in Sweden. Scand Educ Res. (2024) 69(2):1–15. 10.1080/00313831.2024.2330932

[54] Fung W-k Chung K-h Cheng R-y. Gender differences in social mastery motivation and its relationships to vocabulary knowledge, behavioral self-regulation, and socioemotional skills. Early Educ Dev. (2019) 30(2):280–93. 10.1080/10409289.2018.1544004

[55] Thompson AE Voyer D. Sex differences in the ability to recognise non-verbal displays of emotion: a meta-analysis. Cogn Emot. (2014) 28(7):1164–95. 10.1080/02699931.2013.875889 24400860

[56] Archambault I Vandenbossche-Makombo J Fraser SL. Students’ oppositional behaviors and engagement in school: the differential role of the student-teacher relationship. J Child Fam Stud. (2017) 26(6):1702–12. 10.1007/s10826-017-0691-y

[57] De Laet S Colpin H Vervoort E Doumen S Van Leeuwen K Goossens L Developmental trajectories of children’s behavioral engagement in late elementary school: both teachers and peers matter. Dev Psychol. (2015) 51(9):1292–306. 10.1037/a0039478 26192040

[58] León J Liew J. Profiles of adolescents’ peer and teacher relatedness: differences in well-being and academic achievement across latent groups. Learn Individ Differ. (2017) 54:41–50. 10.1016/j.lindif.2017.01.009

[59] Jago R Baranowski T Baranowski JC Cullen KW Thompson DI. Social desirability is associated with some physical activity, psychosocial variables and sedentary behavior but not self-reported physical activity among adolescent males. Health Educ Res. (2006) 22(3):438–49. 10.1093/her/cyl107 16987942

[60] Frøyland LR. Ungdata—Lokale ungdomsundersøkelser. Dokumentasjon av variablene i spørreskjemaet. NOVA. (2017):13–7. Available online at: https://scholar.google.com/scholar?hl=no&as_sdt=0,5&cluster=4273700795942227966

